# Recognition and home care of low birth weight neonates: a qualitative study of knowledge, beliefs and practices of mothers in Iganga-Mayuge Health and Demographic Surveillance Site, Uganda

**DOI:** 10.1186/1471-2458-14-546

**Published:** 2014-06-02

**Authors:** Elizabeth L Nabiwemba, Lynn Atuyambe, Bart Criel, Patrick Kolsteren, Christopher Garimoi Orach

**Affiliations:** 1 School of Public Health, Makerere University College of Health Sciences, P. O. Box 7072, Kampala, Uganda; 2 Institute of Tropical Medicine, Nationalstraat 155, B-2000 Antwerp, Belgium

## Abstract

**Background:**

Neonatal mortality has remained persistently high worldwide. In Uganda, neonatal deaths account for 50% of all infant deaths. Low birth weight is associated with a higher risk of death during the neonatal period. Failure to recognize low birth weight and inappropriate home care practices increase the risk of morbidity and mortality in this high risk group. This study explored mothers’ knowledge, beliefs and practices in recognising and providing home care for low birth weight babies.

**Methods:**

The study was carried out in Eastern Uganda. In-depth interviews were conducted with sixteen mothers of small babies who delivered in health facilities (10) or at home (6) two months prior to the study. Interviews were conducted in mothers’ homes using the local language. Interviewer notes and audio recordings were transcribed and translated to English. Content analysis was done using Atlas-ti software.

**Results:**

Recognition of low birth weight by mothers when a baby is not weighed was difficult. Mothers were aware of the causes of low birth weight though some mothers believed in the influence of supernatural powers. Mothers who delivered in hospital had better knowledge of appropriate home care practices for low birth weight babies compared to mothers who delivered at home or in a lower level health facility. Practices related to cord care and keeping the baby warm were good while poor practices were noted concerning initiation and exclusive breast feeding, and bathing the baby. Low birth weight was not appreciated as a danger sign in newborns and therefore mothers did not seek health care. Some mothers who initiated good care practices for low birth weight newborns in the facilities did not sustain them at home.

**Conclusions:**

Recognition of low birth weight is still poor. This leads to inappropriate home care practices for these high risk newborns. Mothers’ knowledge and care practices can be improved through health education, and this should be extended to the community to reach mothers that deliver at home. Mechanisms to support mothers to sustain good practices should be put in place by taking advantage of existing village health teams and social support.

## Background

Neonatal mortality has remained persistently high worldwide. In Sub-Saharan Africa, where the greatest proportion of neonatal deaths occurs, neonatal mortality rate (NMR) is 35/1000 live births [1]. In Uganda, NMR decreased from 36 to 27/1000 live births between 2001 and 2011, while IMR decreased from 88 to 54/1000 live births. This disproportionate decrease in IMR and NMR resulted into an increase in the proportion of infants that die during the neonatal period from 40% to 50% over the same time period [2]. It is evident that Millennium Development Goal 4 may not be achieved unless deliberate efforts are directed towards reducing deaths during the neonatal period. The majority (75%) of neonatal deaths occur in the first week of life, with 25%-45% of these occurring within the first 24 hours after birth [3]. This points to the need for appropriate care of these babies especially during the critical first days of life. The leading causes of neonatal mortality are complications of preterm births and low birth weight, intra-partum related complications and infections namely sepsis and pneumonia [4]. Inappropriate newborn care practices which increase the risk of neonatal morbidity and mortality are still common in low income countries irrespective of whether the delivery occurred in a health facility or at home. These practices include bathing the baby immediately after birth, giving pre-lacteal feeds, delayed initiation of breast feeding, application of harmful substances on the cord and failure to keep babies warm [5,6].

Low birth weight (LBW), defined as a weight less than 2500 g at birth, is a result of either prematurity or intrauterine growth restriction. LBW has for long been associated with a higher risk of death during the neonatal period because these babies are prone to birth asphyxia, hypothermia, hypoglyceamia due to inadequate feeding and infections e.g. septiceamia [7]. In Uganda NMR is higher among small and very small babies (38/1000 live births) than in normal weight babies (23/1000 live births) [2]. Premature babies continue to die in low income countries due to absence of feasible and cost-effective care, for example keeping warm, breast feeding support, basic care for infections and breathing difficulties [8].

Newborns, especially when LBW or premature are at risk of hypothermia which makes a significant contribution to newborn morbidity [9]. LBW babies can be kept warm by immediate drying and wrapping in warm clothes after birth, frequent feeding, delayed bathing and Kangaroo Mother Care (KMC) [10]. Hypoglyceamia is also a known complication of LBW, although data on its incidence and impact is scarce in low income countries. The most cost-effective strategy in prevention of hypoglyceamia is early initiation and frequent breast feeding [11].

High risk LBW babies need to be identified early and be given the appropriate care to enhance their survival. The number of deaths among LBW could be reduced with low cost interventions that focus on keeping the baby warm, good hygiene, breast feeding support, early identification and management of illness in the first days and weeks of life [12,13]. Mothers and other caretakers play a significant role in the care of newborns, and should therefore have the appropriate knowledge and skills to identify LBW babies and give them appropriate care.

**Figure 1:**
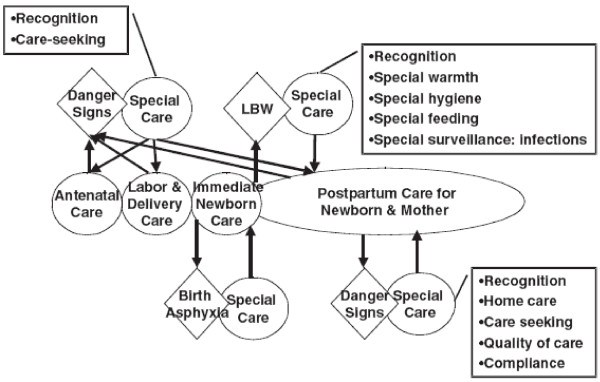
**The figure shows the newborn pathway to survival.** ○-circles represent routine care during each of the four main periods from conception to infancy which is the optimal pathway to health and survival. ◊- detours from the desired pathway. These include maternal danger signs in three settings and three principal scenarios leading to neonatal morbidity and mortality: birth asphyxia, LBW, and development of danger signs, particularly those of infection. □- special care required for mothers and infants who depart from the pathway to survival, emphasizing the fundamental importance of recognition of and appropriate response to illness, including timely care seeking, in the pathway to health maintenance and survival.

Marsh et al. [14] developed a conceptual framework for household and community maternal and newborn care, which highlights elements of newborn care that have been overlooked by safe motherhood and child survival programs. The framework proposes five pathways to improving newborn health and these include 1. use of routine maternal and newborn care services, 2. response to maternal danger signs, 3. response to the non-breathing newborn, 4. care of the low birth weight baby and 5. response to newborn danger signs. According to this framework, care for LBW starts with recognizing them, then they are given special warmth, feeding, hygiene and surveillance for infections (Figure 1). In Iganga where almost half of deliveries occur outside health facilities and therefore babies are not weighed at birth, it is not known if mothers know that their baby has a low birth weight. It is also not known if mothers perceive LBW as a danger sign that requires taking the baby to a health facility or whether they know how to care for them at home. Although there is a considerable amount of research carried out on care of neonates in general [15], there are still few studies focusing on the perceptions about low birth weight and on how mothers care for these high risk babies especially in resource limited settings where much of the care is given at home. This study explored mothers’ knowledge and beliefs in recognizing LBW as well as their home care practices for LBW babies.

## Methods

The study was carried out in Iganga-Mayuge Health and Demographic Surveillance Site, Eastern Uganda. The Iganga-Mayuge HDSS is one of the 33 members of the INDEPTH NETWORK of demographic surveillance sites all over the world. The network covers 18 different countries with 22 sites in Africa. Iganga-Mayuge DSS was established in 2004 with funding from Sida/SAREC and the Rockefeller Foundation. It collects key demographic data which includes pregnancies, births, deaths, migrations, relationships, age, health related information, education among other things. The site covers 3931 square km, is made up of 65 villages and its population has grown to 79,910 as of 30^th^ September 2013. Iganga and Mayuge districts are predominantly rural and the people mainly depend on subsistence farming. The area is served by one hospital and 12 lower level health centres. About 70% of the population lives within five kilometres of a health facility and 67% of deliveries occur in a health facility. Neonatal and infant mortality rates are 23 and 61 per 1000 live births respectively in this region of Uganda.

The study population comprised of mothers of low birth weight or small babies, who had given birth within a period of 2 months prior to the study. We identified the mothers with the help of community health workers who worked in the villages and knew the mothers well.

This was a qualitative study and data were collected through in-depth interviews. Stratified purposeful sampling technique was used to select a total of 16 mothers. According to the prevalence of LBW, we predetermined a sample size of 20 mothers in the one month study period. We aimed at getting an equal number of mothers from two strata according to place of delivery i.e. 10 mothers who delivered in a health facility and 10 mothers who delivered either at their home or at a traditional birth attendant’s (TBA) home. For the later strata we were only able to get 6 mothers of LBW babies who we were certain of because of their small size. Mothers whose babies had not completed the neonatal period of 28 days were excluded from the study. Two female research assistants were trained in conducting in-depth interviews. The research team interviewed the mothers in their homes using the local language and the interviews lasted between 50 and 70 minutes. The interviewers took notes and also tape recorded the interviews with the permission of the respondents. We held meetings at the end of each day to review the transcripts.

The interviewers’ notes and audio recordings were transcribed and translated to English. They were then typed, labeled and stored as MS-word documents. The authors read through the notes several times to get to know the data. The directed approach to content analysis was used to analyse the data. Using this approach, either prior research findings or theory are used to identify key concepts or variables as initial coding categories. In this study we used prior research findings to predetermine initial coding categories. Examples of coding categories generated are hand washing, cord care, feeding, clothing, bathing, danger signs, infections, chronic diseases, maternal nutrition, baby size, etc. We coded relevant text using the predetermined codes and other text that did not fit into the initial coding scheme were given new codes [16]. The new categories generated included; supernatural powers and house heating.

Literature on newborn health shows that morbidity and mortality among LBW depends on recognition of LBW, facility and home care for the LBW newborns (special feeding, special warmth, special hygiene), surveillance for infections and appropriate care seeking [14]. Out of these areas we came up with four themes which are recognizing LBW, causes of LBW, home care practices for LBW newborns (breastfeeding, thermal care, hygiene) and care seeking. We then summarized the category headings under the identified themes namely; recognizing LBW, causes of LBW, home care practices for LBW newborns (breastfeeding, thermal care, hygiene) and care seeking. Two independent persons coded the data and there was significant agreement. Where they disagreed, they discussed and harmonized their views on the codes. Data were analysed using Atlas-ti software.

This study was approved by Makerere University School of Public Health Institutional Review Board and Uganda National Council of Science and Technology. Written informed consent was obtained from the study participants before conducting the interviews.

## Results

Here we report findings of mothers’ knowledge and beliefs concerning recognition of LBW, causes of LBW, and home care practices in four important areas namely; breastfeeding, thermal care, hygiene; and care seeking for LBW babies.

### Characteristics of respondents

Study participants’ ages ranged from 18 to 47 years. Ten mothers had delivered in a government health facility, four at home and two at a TBAs home. Seven mothers were uniparous while nine were multiparous. The mothers belonged to the following religious beliefs; Islam (8), Protestant (5) and Roman Catholic (3).

### Recognition of LBW by mothers

#### Knowledge of how to recognize LBW

Mothers’ recognition of LBW was subjective. In this study mothers used two parameters to recognize low birth weight and these were comparison to size of previous baby and weighing by a health worker. Although multiparous mothers could tell a small baby by comparing the size of the baby with their siblings, uniparous mothers found difficulty in telling whether their baby was small or not. In addition mothers did not know the cut off weight for LBW, so even when the baby’s weight was less than 2500 g the mothers did not know and they were told by a health worker.

Most of the mothers interviewed reported recognizing that their baby was very small immediately after birth, and for the majority their reference point was the size of the previous baby as noted from the quotes below

*“I compared his size to the size of babies I delivered earlier and I could see that he was much smaller than them”* (home delivery, multiparous mother).

*“I noticed she was very small compared to the other children I gave birth to previously”* (facility delivery, multiparous mother).

Some mothers however did not see any problem with their babies, and only learnt that the baby was low birth weight after being told by the midwife. Such mothers were mainly uniparous and the baby’s birth weight was close to 2500 g.

“*I didn’t notice it myself, it was the midwife who told me that Purity had weighed 2.4kgs and that is when I was advised to tie her on my chest so that she could get warmth from me”* (facility delivery, uniparous mother).

#### Beliefs concerning LBW

Beliefs regarding LBW differed between mothers who delivered at home and those who delivered in a health facility. Mothers who delivered at home believed that LBW babies are just like any other baby and do not need special care unless the baby appeared weak and inactive. They believed that physical inactivity rather than birth weight was the pointer that the baby is not normal. On the contrary mothers who delivered in health facilities believed that LBW babies are delicate, prone to illness and need special care even when they do not appear unwell.

*“to me he was normal, in fact when I pushed, he came out and started crying like any other normal baby. Even when my friends came, they insisted that my baby was not normal but I emphasized to them that after pushing him out, he was able to cry. It was only after some days when he become weak and developed chest problems and I took him for treatment to a nurse running a drug shop over there that I believed them”* (home delivery, uniparous mother).

### Causes of LBW

#### Knowledge of causes of LBW

Mothers were aware of the causes of LBW. They mentioned possible causes of LBW e.g. diseases affecting the mother during pregnancy like malaria, syphilis and tuberculosis; pre-maturity; not feeding well during pregnancy and excessive fluid in the womb. Malaria and pre-maturity were the most commonly mentioned causes of LBW

*“I was unwell throughout the pregnancy. I suffered from malaria and syphilis, and I also had no appetite so I wasn’t eating enough food”* (facility delivery, multiparous mother).

*“my baby was small because he was born before the due date, I delivered him at 7 months”* (facility delivery, uniparous mother).

*“I had a discharge of fluid for 3 days before I got labour pains ….. after I realised that I had discharged a lot of fluids I concluded that my baby was very small”* (home delivery, multiparous mother).

A few mothers were not aware of any causes of LBW.

#### Beliefs related to causes of LBW

Beliefs that LBW is caused by supernatural powers and physical phenomena e.g. earth quakes were noted, but in only a few cases. Some believed that earth tremors cause premature labour and hence small babies. Two mothers cited supernatural influences associated with their small babies.

*“people told me that I had reached menopause and the tiny baby because this was the last egg in my womb”* (home delivery, multiparous mother).

*“some people have been saying that the only problem with my baby are the magic powers from a certain* ***big tree with five fingers*** *that I encountered when I was pregnant. I followed their advice and visited a traditional healer with all that was required but there has been no improvement. That is why I decided that I shall only go to hospital”* (home delivery, multiparous mother).

*“my in-laws told me that when my husband lost his mother, they had not given her food prior to her death- so that is why this baby was born very small. They told us that we must go and feed the ‘late mother’ so that this baby can gain weight. This is the advice they gave us but because my husband believes in God he didn’t agree with them”* (facility delivery, multiparous mother).

### Home care practices for LBW babies

The main home care practices for LBW that we were interested in were breastfeeding, thermal care, hygiene and health seeking behaviour. Mothers who delivered in the hospital were aware of what should be done in the areas of feeding, keeping the baby warm and good hygiene. They reported that they were told by health workers that LBW babies are delicate and need special care. However, knowledge of appropriate practices among mothers who delivered in lower level health facilities, at home or at a TBA’s home was low. Beliefs and practices were also associated with place of delivery, with appropriate practices among mothers who delivered in the hospital. On the whole, most mothers were able to practice what the health workers told them to do though a few faced difficulties with exclusive breast feeding when the breast milk seemed not to be enough and doing house chores with the baby tied in the chest. For each of the home care practices, we present results of mother’s knowledge, beliefs and practices.

#### i) Breastfeeding

Although mothers were aware that breastfeeding should be started as soon as possible, they did not know the correct timing. They also knew that LBW babies should breastfeed on demand and not be given any other fluids because the breast milk was sufficient. Only mothers who delivered in health facilities knew that LBW babies may be unable to suckle and that breast milk can be expressed and given with a spoon or through a naso-gastric tube.

Inspite of the awareness of the importance of exclusive breastfeeding, most mothers believed that LBW babies need additional foods to enhance weight gain. Mothers who delivered at home believed that breast milk goes bad when it is outside the breast and is therefore not good for the baby.

For all the mothers who delivered in a health facility and the baby was preterm, breast feeding was initiated after more than 3 hours because the baby had to be taken away from the mother for resuscitation. Even in cases when the baby was not separated from the mother, breast feeding was initiated after several hours because the mothers felt that there was no milk in the breast as yet. The delay in initiating breastfeeding was more common with facility deliveries. Exclusive breastfeeding was practiced especially among facility deliveries. Most mothers reported that for babies who could not suckle from the breast, the milk was expressed and given with a spoon. This practice was taught and demonstrated in the health facilities.

*“these twins did not breast feed soon after delivery so instead I squeezed milk into a cup and used a spoon to feed them…… I did this for two weeks after which I stopped because they had started breast feeding on their own”* (facility delivery, multiparous mother).

*“I was squeezing the breast milk into a cup. I was shown how to feed the baby through a tube that was inserted through the nose. I was instructed to suck the breast milk using a syringe and then push into the tube”* (facility delivery, uniparous mother).

Giving other fluids especially water with glucose before initiating breast feeding was done in some facilities and in home deliveries when the baby was not able to suckle.

*“immediately after delivery the baby failed to suckle the breast and he was crying all the time. I think it was because of hunger, so I gave him water with glucose on the first day. He only started breastfeeding on the second day”* (home delivery, uniparous mother).

Mothers also reported that babies were given either millet or soya porridge in addition to enable them gain weight quickly. This was advice from relatives.

“*my relatives told me to buy millet flour and make porridge for the baby and add blue band [margarine] to the porridge, so that he gains weight quickly. That is what I have been giving him”* (TBA delivery, multiparous mother).

#### ii) Thermal care

All mothers mentioned that LBW babies should be kept warm, but how this is done differed with place of delivery. Irrespective of place of delivery, mothers knew that babies must wear a cap, socks and be wrapped in warm clothes. Only mothers who delivered in the facilities knew that the baby should not be bathed immediately after delivery but they did not know when to start bathing.

*“I didn’t understand the one of bathing the baby; I didn’t know after how many days to start bathing the baby”* (facility delivery, uniparous mother).

Only mothers who delivered in the hospital knew that these babies are put in the mother’s chest to draw warmth from the mother and that this was referred to as kangaroo mother care (KMC).

Most mothers believed that cold air makes the babies head and stomach swell; and also causes vomiting, so they covered the babies to prevent these problems. Mothers also believed that it is not bad to bathe the baby immediately after birth as long as warm water is used.

To keep the baby warm, most mothers said that they dressed the baby in warm clothes, cover the head with a cap and the feet with socks, and then wrap them in sheets and a blanket. They also kept the babies inside the house to prevent exposure to cold air. Some mothers were advised by friends and neighbours to warm the baby using charcoal stoves or jerry cans (plastic 20 liter containers) of warm water as mentioned;

*“when my friends came to see me, they advised that I place a charcoal stove near the baby so that the heat it gives off can provide warmth for him. Others told me to boil water and put in jerry cans and then place them in the room where the baby is so that the warmth can spread to him”* (TBA delivery, multiparous mother).

All mothers who delivered in health facilities did not bathe the baby in the first 24 hours. Mothers who delivered at home or with a TBA bathed the babies within 3–5 hours to clean the baby. There was however no standard practice on how long bathing was delayed. One mother mentioned that due to pressure from other people she bathed the baby earlier than instructed, while others used their own judgement.

“*well I was told not to bathe her for the first two weeks and to keep her indoors but because of pressure from my friends, I bathed her on the fourth day and there was nothing wrong that happened. In fact that day I bathed her, the umbilical cord fell off”* (facility delivery, uniparous mother).

Some mothers reported tying their babies in their chests (KMC) as shown in the health facilities, while others said they could not because they were only given verbal instructions on how to do it. None of the mothers who delivered at home or with a TBA practiced KMC.

*“the midwife showed me how to tie the baby in my chest. She first undressed the baby and then placed her onto my bare chest like this and then tied a cloth across to my back. I also make sure there is always a cap on her head”* (facility delivery, multiparous mother).

*“I was just told to tie the baby in the chest, I was not told not to bathe the baby… when I returned for review the baby’s weight was less than at birth so the doctor admitted the baby to show me the correct way of putting the baby in my chest….the baby stopped oversleeping and became active. At discharge he had gained weight”* (facility delivery, uniparous mother).

#### iii) Hygiene

Most mothers mentioned proper hygiene as keeping the cord clean, changing baby’s clothes when they are soiled, and keeping the feeding cups clean. None of the mothers mentioned hand washing with soap before handling the baby.

Concerning keeping the cord clean, most mothers reported cleaning the cord with warm water with salt added. Most mothers who delivered in health facilities did not apply anything, while a few admitted to putting surgical spirit and these were all multiparous mothers. The practice of applying powder on the cord was found among home deliveries.

*“I used to clean it with a small piece of cloth soaked in warm water with salt then after which sprinkle powder”* (home delivery, multiparous mother).

Mothers also reported immediately changing the baby’s clothes when they are soiled. Hand washing was not a common practice and a few who did it mentioned that they wash hands only if they feel that the hands are very dirty for example after doing housework or farming.

#### iv) Health care seeking

All mothers knew that LBW babies should be taken to hospital for proper examination when they fall sick.

*“such babies must be taken to hospital for tests to establish the actual illness before treatment is administered”* (facility delivery, multiparous mother).

However mothers did not know that LBW babies born at home should be taken to a health facility for initial assessment and regular growth monitoring.

For mothers who delivered at home or TBAs, none of them took the baby to a health facility to be examined by a health worker. When asked why they didn’t go, they responded that they did not think that it was necessary since the baby looked well.

Although mothers knew that LBW babies should be taken to hospital for treatment, some mothers sought care from drug shops and clinics when they felt that the illness was mild.

Mothers who were advised to return to the health facilities to have their baby’s growth monitored complied most of the time.

*“I was told by the midwife to take the baby back every week to monitor his weight and health. Whenever I took him back I found that his weight had increased and this encouraged me”* (facility delivery, uniparous mother).

However mothers who delivered at home and did not receive any advise on seeking care did not take the babies for assessment and growth monitoring.

*“no one told me to take the baby to hospital to be examined and weighed, so I did not go since the baby had no problem”* (home delivery, multiparous mother).

#### Challenges to home care of LBW

The main challenges mothers found while caring for LBW babies were not giving other foods, not bathing the baby, maintaining KMC and keeping indoors. Some mothers reported that it was not easy to give the baby only breast milk without additional drinks.

*“I was even advised not to give the baby other foods until she is 6 months old. But sometimes the breast milk is not enough for her but I can’t do much because I can’t give her any other food, so I am inconvenienced because she keeps crying”* (facility delivery, uniparous mother).

Mothers reported that they did not tie the babies to the chest all the time as instructed by the health workers. Several mothers found tying the baby in the chest very tiring, with some indicating that it caused chest pain and backache. A mother of twins narrated

“*they are so demanding in terms of placing them onto my chest and once in a while I feel very tired….. occasionally I experience backache since I was operated and also the stitched area pains me a lot but I will be fine”* (facility delivery, multiparous mother).

In some instances the husband relieved the mother by either tying the baby in his chest or helping with other work e.g. washing clothes. Mothers also felt that not bathing the baby was unusual, and that it caused discomfort to the baby hence the baby cries all the time. Mothers indicated that it was difficult to be indoors with the baby all the time

*“I find it boring to keep in doors all the time….. the health worker told me it is not good to move around with this baby for everybody to touch and see, so I am always inside the house”* (facility delivery, multiparous mother).

Some of the mothers found it difficult to send away visitors without them seeing the baby. They felt it was not proper in their culture.

## Discussion

The study findings show that recognition of LBW by mothers is difficult especially when the baby is not weighed. Knowledge regarding causes and care of LBW babies was high among mothers who delivered in the hospital compared to those who delivered in lower level health facilities or at home. There are beliefs especially associated with causes of LBW, keeping the baby warm and feeding which affect the home care practices either positively and negatively. Home care practices were good in some aspects like cord care but poor regarding hand washing with soap. Mothers who delivered in the hospital had good home care practices inspite of some challenges.

### Recognition of LBW by mothers

From this study we find that mothers do not know that a birth weight below 2.5 kg is low birth weight. However mothers judged a baby as being small in comparison to the size of their previous babies. This indicates that for first time mothers, it may be difficult to tell whether their baby is LBW since they have no other baby to compare with, and this is even more difficult when the weight is border line. But also if a mother has been giving birth to small babies, she may believe that is the normal size of newborns and therefore miss identifying LBW. In situations where babies are not weighed at birth, identifying LBW is then purely subjective and if it is left to mothers to decide, then many LBW will miss getting essential care because they are not identified.

In addition to recognizing a LBW baby, it is also crucial for the mother to understand that LBW is a danger sign, and is therefore a reason for seeking care. In this study mothers who delivered at home, did not feel that being small was a problem except when the baby appeared weak. This is in contrast to those who delivered in the facilities who were told by health workers that LBW babies are delicate and need special care. These results agree with findings in a rural Indian community which showed that birth weight per se was not considered a determinant of newborn health by the mothers, but mothers classified newborns basing on signs like level of feeding, vigour and alertness [15]. Therefore a baby who has a low birth weight but does not have any signs that cause concern to the mother is not likely to be taken to a health facility, which is a missed opportunity for the mother to receive health education on appropriate home care practices for LBW. This calls for the need for interventions for newborns to emphasise to mothers that LBW babies are vulnerable to poor health and need special care irrespective of presence or absence of any visible signs.

### Causes of LBW

In this study, mothers generally were aware of the causes of LBW. The main causes mentioned were malaria, syphilis, poor feeding and excessive fluids in the uterus. What mothers believe to be the causes of LBW will affect what they will do to prevent it in future pregnancies. Mothers could be helped to prevent LBW in future by giving them health education on proper nutrition during pregnancy, family planning to improve on birth spacing and on prevention of diseases like malaria. This would reduce the proportion of vulnerable neonates and in the long run reduce neonatal mortality. Mothers with false beliefs would also benefit from the correct information during health education.

### Home care practices for LBW

The main home care practices were in the areas of breastfeeding, thermal care, hygiene and health care seeking. The study found that mothers who delivered in hospital were aware of the appropriate homecare of LBW in comparison to those who delivered in lower level health facilities or at home. This could be explained by the fact that midwives in the hospital were trained in newborn care and were therefore giving health education to the mothers, unlike midwives in other facilities that were not trained. Mothers who delivered at home and did not receive any health education had very low knowledge of how to care for LBW babies. The study findings show that the level on knowledge was associated with the care practices. Mothers who knew what to do also practiced it, although in some instances they did not do it correctly. This could have been that the instructions were not clear or the mothers faced other challenges at home.

As in previous studies, this study shows that inappropriate homecare practices are still prevalent in the community and these include delayed initiation of breast feeding, giving pre-lacteal feeds, early bathing, poor hygiene and exposure to cold [17-19]. Therefore interventions for LBW must include health education to emphasise these areas.

It is recommended that breastfeeding must be initiated within the first hour after birth, but this was a challenge even within health facilities. This was because most LBW babies were preterm and they required resuscitation which necessitated separating the baby from the mother. However, this can be improved by ensuring that health workers return the baby to the mother as soon as they are stable to enable breast feeding. It was commendable that expressing breast milk was done among facility deliveries as this promotes milk production and reduces chances of giving other feeds to the LBW newborn. There was a general belief especially among mothers who had not received health education that LBW babies need additional feeds to enhance weight gain and that mothers cannot produce enough breast milk to satisfy them. This led to the bad practice of early introduction of supplementary foods. Mothers need to be reassured that breast milk alone is sufficient even for the LBW baby’s nutritional requirements so that they are not compelled to supplement with other feeds. A study that compared exclusive breastfeeding LBW babies to partially breastfed and non-breastfed LBW showed that the increase in weight and length did not differ between the feeding groups. In addition the partially breastfed and non-breast fed had more frequent and more severe episodes of respiratory infections when compared to the exclusive breastfeeding group [20]. So during health education mothers must be assured that in the first six months of life, breast milk alone is sufficient for proper growth and protection against infection in LBW neonates.

Low birth weight babies were generally viewed as being vulnerable to cold because of their small size. The protective measures mentioned in this study were similar to those mentioned in other studies e.g. confining them indoors, covering them, placing them near objects heated by fire [21]. In this study we find that the information given to mothers concerning delay to bathe the baby is incomplete and/or unclear. When this is so then when to start bathing the baby depends on the mother’s judgement and this could lead to unnecessary exposure to cold. Health workers must give clear guidelines on when the baby should be bathed so that mothers do it at the right time.

A meta-analysis showed that KMC reduces neonatal morbidity and mortality in pre-term babies [12]. In this study most mothers were willing and some had even tried it, but the major challenge to skin-to-skin care is the lack of continuity. In Ghana, mothers reported that backache, time constraints, fear to harm the cord were barriers to skin-to-skin care and the practice was cautiously adopted [22]. In this study, mothers that delivered in the hospital were told about KMC, although it was not demonstrated in all the cases except for babies who spent some days in the KMC room, where health workers showed the mothers how to correctly tie the baby in the mother’s chest. Since KMC is relatively new, health workers should demonstrate to mothers how to do it correctly. Mothers will most likely not practice KMC if they do not feel confident enough that they are doing it right.

A major barrier to care seeking is failure or delay to recognize danger signs in the neonate by the mother or caregiver [23]. This was also found in our study whereby mothers did not perceive LBW as a danger sign and they did not seek care outside the home. Although mothers kept LBW babies in the house most of the time, restriction of movement of the newborn was not found to be a hindrance to care seeking when the baby was ill or needed to be taken to a health facility for follow-up. This was similar to findings of studies in Tanzania and Bangladesh although in these studies mothers were constrained by some barriers such as lack of money for transport and treatment [21,24].

This study revealed that mothers had beliefs and practices regarding causes and care of the LBW that were driven by their culture. This is in agreement with a study in India about newborns in general which showed that mothers had cultural beliefs and practices regarding cord care, giving colostrum to the baby, when to take the baby out of the house and weighing the baby frequently [25].

### Challenges to home care of LBW

This study identified challenges with homecare of LBW in relation to feeding and keeping the baby warm. Although caring for LBW babies comes with challenges, with adequate health education mothers are able to overcome the challenges for the sake of their baby’s survival. Most mothers had intentions to fully comply with the pieces of advice given by the health workers on exclusive breast feeding, delay to bathe, keeping the baby indoors and taking the baby to a health facility for growth monitoring, but sometimes they could not. For example mothers felt that exclusive breast feeding was not sufficient for small babies and they at times gave additional foods to enhance weight gain. There is evidence that LBW babies are able to gain weight as expected when they are fed on breast milk alone and therefore do not require supplementary feeding before 6 months. Nutrition education is useful in reinforcing the practice of exclusive breast feeding, thus reducing the risk of malnutrition and mortality in LBW babies [26]. According to the conceptual framework by Marsh et al. [14], care for LBW begins with recognizing them. This study shows that this is still a challenge if the baby is not weighed because there are currently no other measures apart from estimating from the size of the baby, which is very subjective. But also equally important is the appreciation that LBW is a danger sign irrespective of the apparent state of the baby, which is still lacking. It was not clear from this study whether and how surveillance for infections among LBW is done.

In Uganda, the VHT system is being scaled to all districts to increase access to health information, health care as well as empowering communities to take charge of their health. The findings of this study can be used to lobby for inclusion of health education and support for LBW in the newborn care package. Given that VHTs work in the community, it is envisaged that there will be continuity of care between health facilities and homes, closer and more regular support to mothers for appropriate home care of LBW babies. VHTs encourage mothers to deliver in health facilities, which will lead to more LBW babies being identified at birth and receiving the appropriate care.

Limitations – this study was based on mothers reported practices rather than on observation. These self reports are prone to recall and response bias, which we tried to minimize by establishing rapport with the respondents and careful probing during the interview. The selection process whereby mothers were identified by CHWs who are known to them may lead to selection bias. The fact that there were more mothers who delivered in health facilities than those who delivered at home, could lead to information bias with a tendency of hearing more from mothers who are likely to be more knowledgeable because of interaction with health workers.

## Conclusions

Recognition of LBW among babies that are not weighed at birth is still a challenge. Mothers are aware of the causes of LBW but there is belief in supernatural influences as well. Mothers are aware of the appropriate home care practices in the key areas of breast feeding, thermal care, hygiene and health care seeking but some inappropriate practices still exist in the community. There are differences in knowledge and home care practices between mothers in this community who delivered in health facilities and those who delivered at home or with a TBA. This is a result of the health education received in the health facilities.

### Recommendations

Mothers should be encouraged to deliver in health facilities where they will receive health education. Family and community support structures should be strengthened and equipped to help promote good practices and discourage inappropriate practices by involving significant others in the community.

Health education should continue in health facilities, but also extend into the community to reach mothers that do not utilize the formal health care system. The health system should take advantage of the existing VHT structures to train and supervise VHT members on how to give health education related to home care and referral of LBW babies. Mechanisms to support mothers to sustain good practices should be put in place through home visits by VHTs.

In order to design behavior change interventions to improve home care practices that are culturally acceptable and effective, researchers should consider beliefs and practices in that specific community concerning birth weight and its implications to the wellbeing of LBW babies and home care practices. In order to reach more LBW babies in the community, the authors suggest community based studies to determine ways of identifying LBW babies who are delivered at home and are not weighed at birth; and giving their mothers health education in newborn homecare practices. These studies would provide more insight on how to improve the health and survival of LBW babies.

## Abbreviations

KMC: Kangaroo mother care; LBW: Low birth weight; STS: Skin-to-skin care; TBA: Traditional birth attendant.

## Competing interests

The authors declare that they have no competing interests.

## Authors’ contributions

NLE was responsible for the conception and design of the study, analysis and interpretation of data, and drafting and revising the manuscript. LA was involved in the design of the study, data collection, analysis and interpretation. He also reviewed and revised the manuscript. OGC contributed to the conception and design of the study, data analysis, interpretation, reviewing and revising the manuscript. KP and BC contributed to drafting, reviewing and revising the manuscript. All authors read and approved the final manuscript. The authors have adhered to the BMC RATS guidelines for reporting qualitative studies.

## Pre-publication history

The pre-publication history for this paper can be accessed here:

http://www.biomedcentral.com/1471-2458/14/546/prepub
